# Pancreatic diffuse large B-cell lymphoma: a case report and literature review

**DOI:** 10.3389/fonc.2023.1294385

**Published:** 2023-11-22

**Authors:** Lijun Shi, Juan Wang, Li Wei, Zhongrui Ma, Xaiohu Liu

**Affiliations:** ^1^ Department of Hematology, Chengdu Fifth People’s Hospital, Chengdu, China; ^2^ Department of Gastroenterology, Chengdu Fifth People’s Hospital, Chengdu, China; ^3^ Department of Psychosomatic Medicine, Chengdu Fifth People’s Hospital, Chengdu, China; ^4^ Department of Radiology, Chengdu Fifth People’s Hospital, Chengdu, China

**Keywords:** pancreatic tumor, non-Hodgkin’s lymphoma, diffuse large B-cell lymphoma, diagnosis, jaundice pancreatic tumor, diffuse large B-cell lymphoma, jaundice

## Abstract

Primary pancreatic lymphoma is an extremely rare malignant tumor that accounts for 1% and 0.5% of all extranodal malignant lymphomas and pancreatic tumors, respectively. The clinical and radiographic characteristics of primary pancreatic lymphoma are non-specific, and it is often misdiagnosed as pancreatic cancer or pancreatic tuberculosis, delaying treatment. The most common histological subtype of primary pancreatic lymphoma is diffuse large B-cell lymphoma. Herein, we report a case of a 48-year-old female patient who was hospitalized for complaints of lower back pain, jaundice, dark brown urine, nausea, and ascites. Radiological evaluation revealed a pancreatic head mass that was diagnosed as diffuse large B-cell lymphoma following ultrasound-guided percutaneous fine-needle biopsy. During hospitalization, the patient’s jaundice worsened, and percutaneous transhepatic drainage was performed. However, hemorrhagic ascites and disorders of consciousness occurred after surgery, and the patient died due to multiple organ failure. Considering the outcome of this case, we reviewed the existing relevant literature on primary pancreatic lymphoma to better understand the disease to facilitate timely diagnosis and initiation of treatment.

## 1 Introduction

Primary pancreatic lymphoma (PPL) is an extremely rare malignant tumor that accounts for 1% and 0.5% of all extranodal malignant lymphomas and pancreatic tumors, respectively (1). PPL is more common in men than in women, and its average age of onset is 53 years (2). The clinical and radiographic characteristics of PPL are non-specific, and PPL is often misdiagnosed as pancreatic cancer or pancreatic tuberculosis. Diffuse large B-cell lymphoma (DLBCL) is the most common (77%–80%) histological subtype of PPL (3, 4). The treatment strategies for PPL include surgery, chemotherapy, radiotherapy, or comprehensive treatment; however, chemotherapy remains the standard treatment option. We report a case of a patient with primary DLBCL of the pancreas who died due to the lack of timely and effective treatment after diagnosis. Considering the outcome of this case, we reviewed the existing relevant literature on PPL to strengthen the understanding of this disease to facilitate the accurate diagnosis and timely initiation of treatment.

## 2 Case report

**Figure 1:**
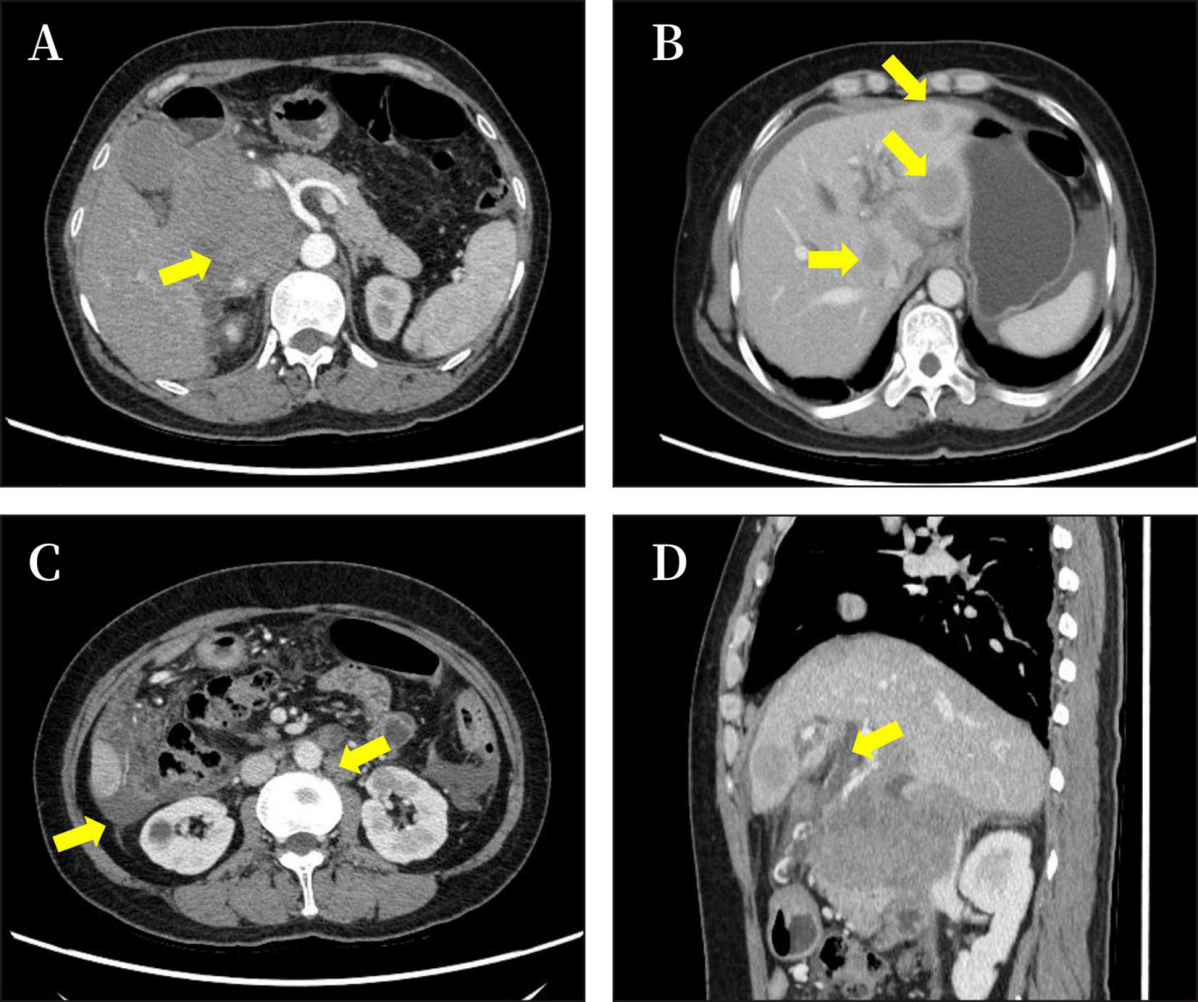
Imaging results of the abdomen. The results of CT showed a pancreatic head mass (size approximately 8.6 × 7.1 cm) **(A)**, with intrahepatic metastasis, ascitic fluid **(B)**; multiple enlarged peripancreatic and retroperitoneal lymph nodes, ascitic fluid **(C)**; and dilatation of intrahepatic bile duct **(D)**.

**Figure 2:**
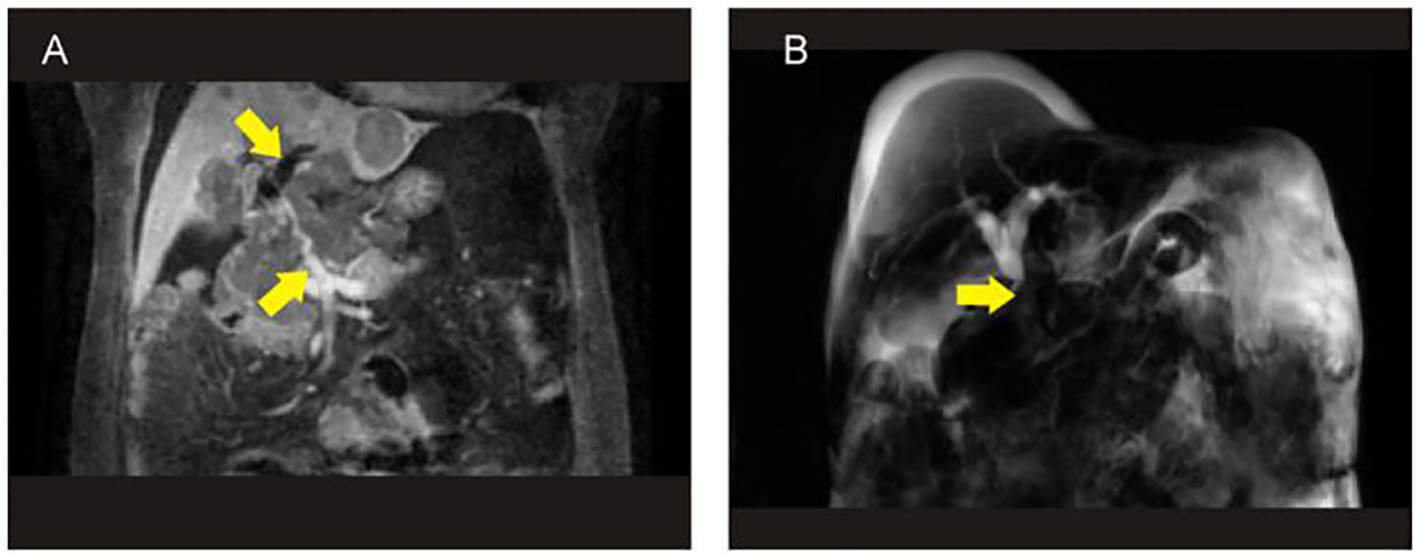
The results of MRI+MRCP showed that the tumor contained blood vessels **(A)** and biliary obstruction **(B)**. MRCP, magnetic resonance cholangiopancreatography.

**Figure 3:**
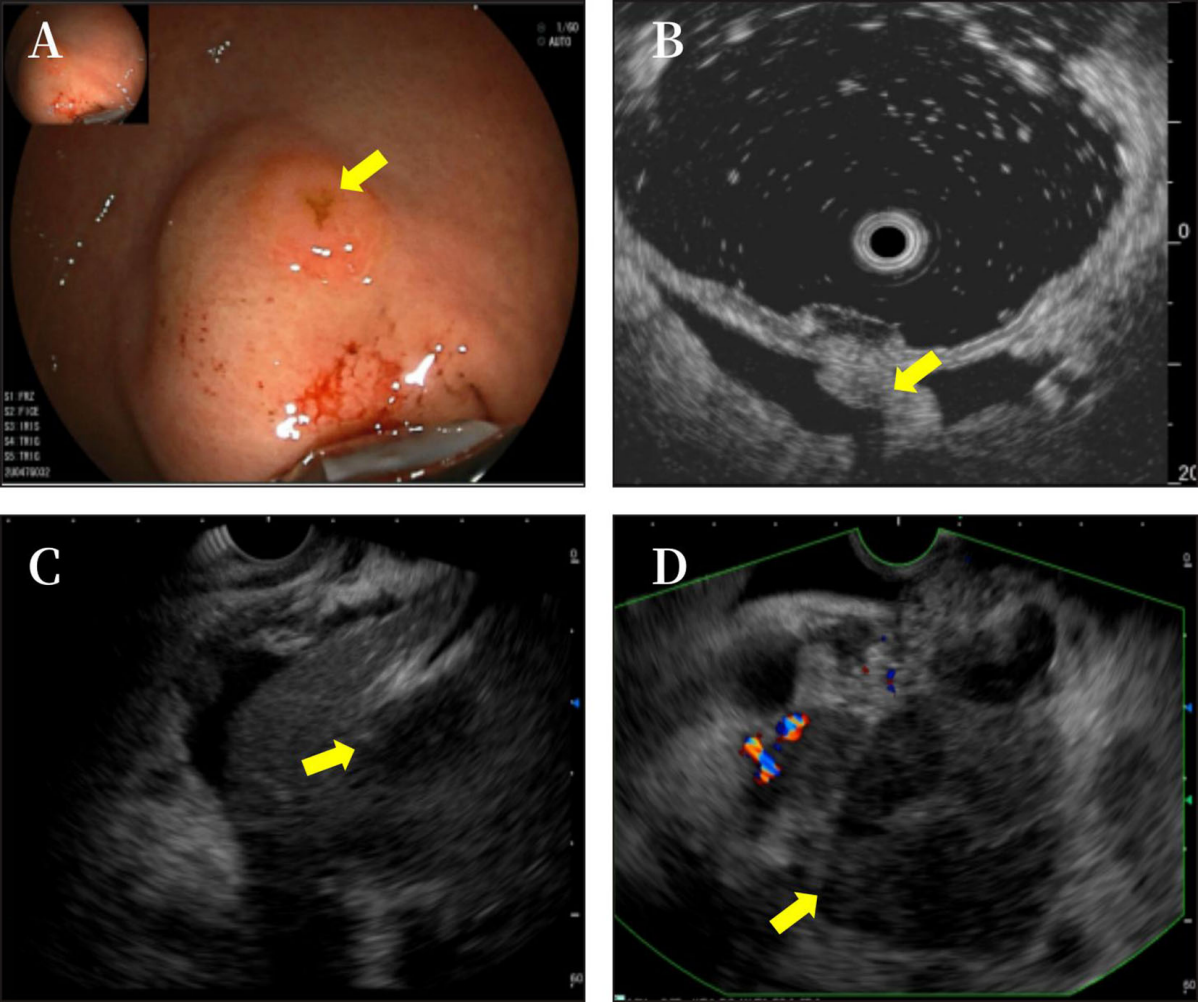
The gastroscope showed a submucosal uplift in the fundus of the stomach **(A)**. Endoscopic ultrasound showed phrenogastric ligament **(B)** and multiple hypoechoic masses in the liver **(C)** and a mixed rising mass in the head of the pancreas with poorly defined boundaries. The measured cross-sectional size of the mass was 4.9 cm × 4.8 cm **(D)**.

**Figure 4:**
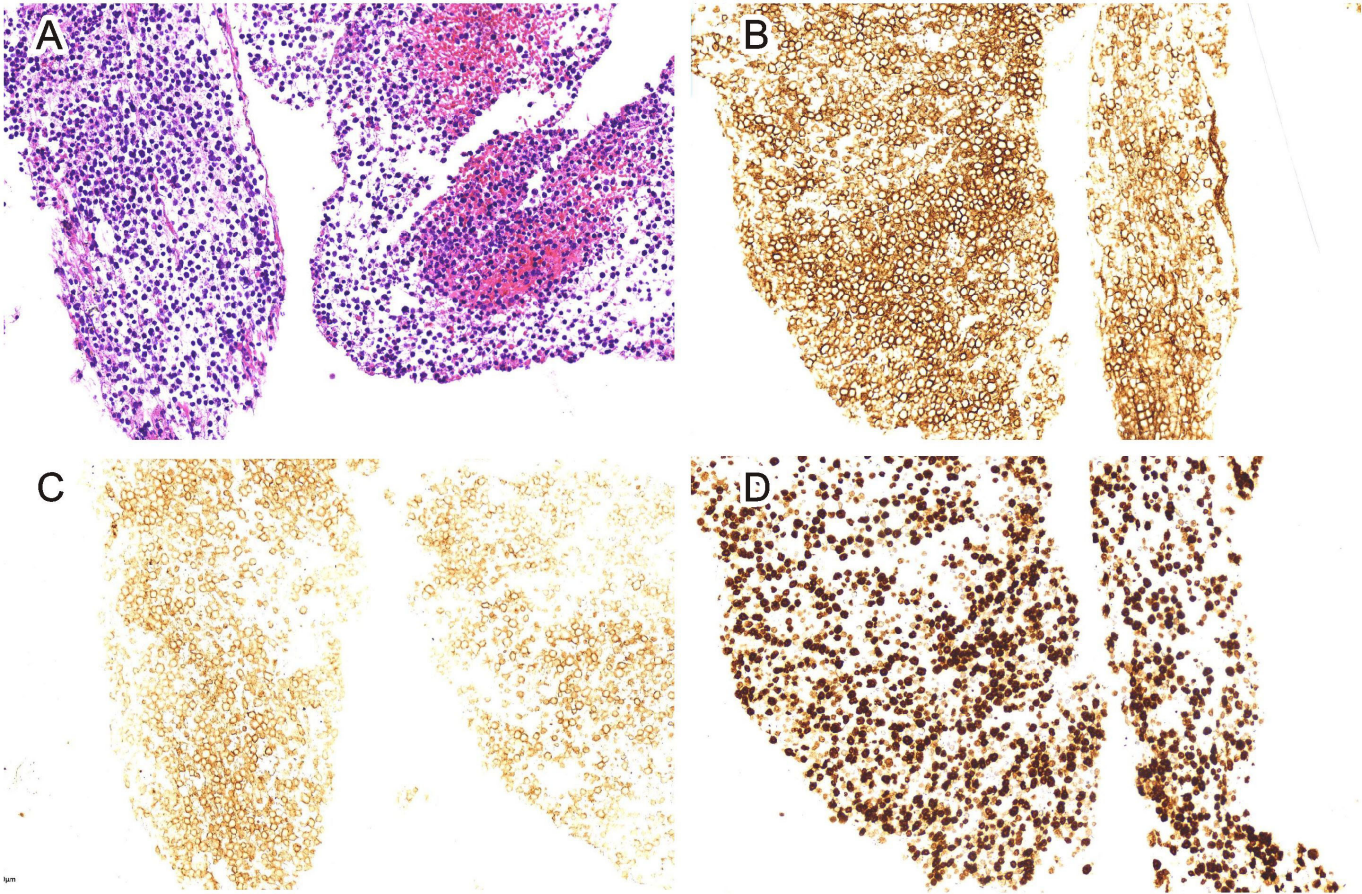
Pancreas biopsy (H&E staining, 20-fold) **(A)**. Immunohistochemical CD20 staining showed positive tissue cells (EnVision staining, 200-fold) **(B)**. Immunohistochemical CD10 staining showed negative tissue cells (EnVision staining, 200-fold) **(C)**. Immunohistochemical Ki-67 staining showed positive tissue cells (EnVision staining, 200-fold) **(D)**.

A 48-year-old woman was admitted to the oncology department of our hospital with complaints of approximately 10-day lower back pain, jaundice, dark brown urine, nausea, and ascites on 4 October 2022. Physical examination revealed no enlarged superficial lymph nodes or palpable masses in her upper abdomen. The patient’s laboratory tests are shown in **Table 1**. Contrast-enhanced abdominal computed tomography (CT) revealed a low-density mass of 8.6 cm × 7.1 cm on the head of the pancreas that involved the portal vein and multiple possible intrahepatic metastases. CT also showed multiple enlarged peripancreatic and retroperitoneal lymph nodes, possible peritonitis, gallbladder cholestasis, dilatation of intrahepatic bile duct, unclear display of extrahepatic bile duct, and fluid in both the abdomen and pelvis (**Figure 1**). Abdominal enhanced magnetic resonance imaging with magnetic resonance cholangiopancreatography revealed a pancreas head mass of approximately 8.8 cm × 6.8 cm in size, neoplastic lesions, local narrowing of the portal vein caused by lesions surrounding it, and multiple intrahepatic metastases. Multiple enlarged peripancreatic and retroperitoneal lymph nodes were identified, and the possibility of metastasis was considered. The gallbladder was slightly enlarged, the intrahepatic and extrahepatic bile ducts were slightly dilated, and the lower segment of the common bile duct was unclear (**Figure 2**). The patient had a pancreatic mass, but the type of the mass was unclear. After obtaining the consent of the patient and her family, an endoscopic ultrasound-guided fine-needle biopsy of the pancreas was performed on 7 October 2022. The gastroscope showed a submucosal uplift in the fundus of the stomach, which was yellow and slightly depressed in the center. Endoscopic ultrasound showed multiple hypoechoic masses in the liver, dilated intrahepatic and intrahepatic bile ducts, and a mixed rising mass in the head of the pancreas with poorly defined boundaries. The measured cross-sectional size of the mass was 4.9 cm × 4.8 cm. The pancreatic duct was not dilated, and the parenchymal echo of the pancreatic body and tail was uniform (**Figure 3**). Preliminary pathological results showed more small lymphocytes, but immunohistochemical results were not returned. After treatment with adenosyl-methionine butanedisulfonate for improvement of jaundice, magnesium isoglycyrrhizinate for liver protection, furosemide and spironolactone diuresis, and cefuroxime for anti-infection, the patient’s jaundice worsened, and she was transferred to the department of gastroenterology on October 9 for endoscopic retrograde cholangiopancreatography (ERCP) treatment. After the transfer, the doctor communicated with the patient and her family and suggested ERCP treatment. However, due to the patient’s poor general condition and the patient’s reluctance to undergo ERCP treatment under general anesthesia, the patient only continued to receive jaundice treatment, liver protection, anti-infection, and other treatments. On October 12, a postoperative puncture biopsy of the pancreatic mass, combined with immunohistochemistry, revealed non-Hodgkin’s lymphoma, consistent with DLBCL of central germinal origin (**Figure 4**). The results of the immunohistochemistry analysis were as follows: Bcl2 (+), Bcl6 (−), CD10 (+), CD20 (+), CD21 (−), CD23 (−), CD3 (−), CD5 (−), CyclinD1 (−), Ki-67 (80% positive), and EBER (−) (**Figure 4**). A diagnosis of pancreatic DLBCL was established based on the histological features and immunophenotypes mentioned above. The treatment plan for the patient included improvement of jaundice, liver protection, and prevention of infection. Reexamination of the patient’s liver function showed that the jaundice had worsened and her direct bilirubin (219.6 mmol/L), total bilirubin (316.9 mmol/L), and total bile acid (12.5 mmol/L) levels had increased compared to the baseline values. Thus, percutaneous transhepatic cholangiodrainage (PTCD) was administered for the treatment of jaundice, and chemotherapy was scheduled thereafter. The doctor communicated with the patient and her family again, suggesting chemotherapy after PTCD for jaundice improvement. The patient and her family agreed to this treatment plan. Unfortunately, the patient developed hemorrhagic ascites after the PTCD treatment. Her overall condition worsened, and she eventually developed disorders of consciousness, which resulted in multiple organ failure and death.

**Table 1:** The patient’s laboratory test results.

	2022/10/4	10/7	10/11	10/12	10/14 am	10/14 pm	10/15
WBC (×10^9^/L)	6.17	5.71	10.95	10.04	12.52	14.69	17.07
ANC (×10^9^/L)	5.2	4.93	9.98	9.08	11.23	15.15	13.16
PLT (×10^9^/L)	162	125	125	137	187	217	113
Hb (g/L)	127	113	130	132	129	99	82
C-reactive protein (mg/L)	6.3	12.2	25.1	24.5	19.1	16.7	
Total bilirubin (mmol/L)	129.8	165.6	280.7	316.9	345.8	194.1	
Direct bilirubin (mmol/L)	89.8	119.2	195.6	219.6	245.3	144.4	
Indirect bilirubin (mmol/L)	40	46.4	85.1	97.3	100.5	49.7	
Total bile acid (mmol/L)	134				221.8	12.5	
Albumin (g/L)	39.6	32.1	33.7	33.5	21.7	30.8	36.5
Alanine aminotransferase (U/L)	183	143	121	141	170		
Aspartate amino transferase (U/L)	170	138	191	236	355		
Alkaline phosphatase (U/L)	247	225	522	923	708		
γ-Glutamyl transferase (IU/L)	715	544					
Lactate dehydrogenase (U/L)	793			1415			42505
β2-Microglobulin (mg/L)	4.3	3.1		17.4	24.5		
Urea (mmol/L)	5.18	3.32	10.09	16.76	26.24		
Creatinine (mmol/L)	83.8	63.3	107.7	146.8	258.1		
Prothrombin time (s)	13.6			32	46.4	48.1	71.3
Activated partial thromboplastin time (s)	28.4			42	48.7	54.4	65.5
INR	1.12			1.99	3.83	4.61	6.07
Thrombin time (s)	16.6			17.1	18.4	18.6	24.7
Fibrinogen (g/L)	2.97			2.91	2.87	2.65	1.05
Cancer antigen (CA) 19-9 (ng/mL)	235.43						
Cancer antigen (CA) 72-4 (U/mL)	0.89						
Cancer antigen (CA) 12-5 (U/mL)	44.1						
Cancer antigen (CA) 15-3 (U/mL)	14.78						
Ferritin (ng/mL)	336.9						
Carcinoembryonic antigen (CEA) (ng/mL)	2.26						
Alpha-fetoprotein (AFP) (ng/mL)	3.03						

WBC, white blood cell; ANC, neutrophils; PLT, platelet; Hb, hemoglobin; INR, international normalized ratio.

## 3 Discussion

PPL, a lymphoma localized to the pancreas with or without peripancreatic nodal involvement, is a rare disease. Older patients and patients with stage IV disease or an aggressive PPL subtype have a worse prognosis (5).

The clinical features of PPL are non-specific and include middle and upper abdominal pain, weight loss, abdominal mass, obstructive jaundice (6, 7), intestinal obstruction, and acute pancreatitis (8), which are similar to the symptoms of pancreatic adenocarcinoma or inflammatory pancreatic disease (9). Typical symptoms of non-Hodgkin’s lymphoma, such as fever and night sweats, are rare in PPL (1). In most patients with PPL, the upper abdominal mass that is characteristic of the disease can be palpated on physical examination. Radiologically, PPL lesions appear as low-density shadows with clear edges and uneven enhancement in the arterial phase, which may manifest as pancreatic duct interruption or stenosis; pancreatic duct dilation or necrosis are usually not present (10, 11). This observation is related to the soft texture of pancreatic lymphoma and the fact that the tumor does not originate from pancreatic ductal epithelial cells, and the pancreatic duct itself is less involved. In contrast, retroperitoneal lymph nodes are often involved. Patients with PPL commonly show normal or slightly elevated levels of tumor markers, such as CA19-9, CA125, and carcinoembryonic antigen, in cases of biliary obstruction, which is valuable for the differential diagnosis of common pancreatic masses. Elevated liver enzymes and bilirubin levels are also observed in patients with PPL and biliary obstruction. Therefore, combined with the clinical manifestations, laboratory examination, and imaging results of this patient, it is considered that PPL may be complicated by obstructive jaundice.

The final diagnosis of PPL depends on pathological biopsy findings, which can be confirmed by ultrasound using CT-guided percutaneous fine-needle puncture biopsy, endoscopic fine-needle puncture biopsy through the posterior gastric wall or duodenum, or surgical exploratory abdominal biopsy (12). In this case, according to pathological immunohistochemical results, the diagnosis of PPL was clear. Considering that DLBCL is the most common histopathological subtype of PPL, the cyclophosphamide, doxorubicin, vincristine, and prednisone regimen or the rituximab, cyclophosphamide, doxorubicin, vincristine, and prednisone regimen is the standard treatment (1). These treatments are generally administered in six courses. The second-line rituximab, isocyclophosphamide, carboplatin, and etoposide regimen or the rituximab, dexamethasone, high-dose cytarabine, and cisplatin (5) regimen can improve the prognosis of patients with refractory pancreatic DLBCL (4, 5). Due to the large size of the tumor and the high risk of postoperative pancreatic fistula, surgical intervention is not the main treatment option for PPL (13). However, if diagnosis of the pancreatic mass cannot be confirmed using non-surgical options or if the patient’s main symptom was caused by biliary obstruction, surgical treatment can be performed. Radiotherapy is often administered as an adjunct to chemotherapy for PPL. Studies have shown that the total effective rate of chemotherapy and radiotherapy is higher than that of chemotherapy alone (6, 14). However, the contribution of radiotherapy to the superior effect of the combined therapy has not been well defined (1). In this case, low-dose dexamethasone can be used to reduce tumor burden after the initially suspected lymphoma is accompanied by a pathological biopsy. After a definite diagnosis of DLBCL of the pancreas, patients with jaundice may be gradually treated, and their condition improved when rituximab plus cyclophosphamide, doxorubicin, vincristine, and prednisone (RCHOP) is given in time. Unfortunately, the doctor in charge of our patient only paid attention to the jaundice of the patient, ignoring the disease itself, and did not consult the hematologist during the whole treatment period. Finally, percutaneous transhepatic cholangiodrainage (PTCD) was chosen for treatment due to aggravated jaundice, and the coagulation function review before PTCD treatment indicated that prothrombin time and activated partial thromboplastin time were prolonged, which may have led to abdominal bleeding after PTCD treatment was accomplished. This is a case worth reflecting on. In future clinical work, we need to think about how to better manage and treat such patients.

In conclusion, it is important that once DLBCL of the pancreas is suspected or diagnosed, systemic chemotherapy is recommended as soon as possible to prevent delay or even irreversible results.

## Data availability statement

The original contributions presented in the study are included in the article/supplementary materials, further inquiries can be directed to the corresponding author.

## Ethics statement

The studies involving human participants were reviewed and approved by Chengdu Fifth People’s Hospital. Written informed consent to participate in this study was provided by the participants’ legal guardian. Written informed consent was obtained from the individual(s)/legal guardian, for the publication of any potentially identifiable images or data included in this article.

## Author contributions

LS: Conceptualization, Data curation, Formal Analysis, Investigation, Methodology, Resources, Writing – original draft, Writing – review & editing. JW: Resources, Conceptualization, Writing – original draft. ZM: Investigation, Writing – review & editing. LW: Conceptualization, Resources, Writing – review & editing. XL: Resources, Conceptualization, Investigation, Writing – original draft.
